# Prescribing Trends in Bipolar Disorder: Cohort Study in the United Kingdom THIN Primary Care Database 1995–2009

**DOI:** 10.1371/journal.pone.0028725

**Published:** 2011-12-07

**Authors:** Joseph Hayes, Philip Prah, Irwin Nazareth, Michael King, Kate Walters, Irene Petersen, David Osborn

**Affiliations:** 1 Mental Health Sciences Unit, University College London, London, United Kingdom; 2 MRC General Practice Research Framework, London, United Kingdom; 3 Department of Primary Care and Population Health, University College London, London, United Kingdom; Federal University of Rio de Janeiro, Brazil

## Abstract

**Objectives:**

To determine changes in prescribing patterns in primary care of antipsychotic and mood stabiliser medication in a representative sample of patients with bipolar disorder in the United Kingdom over a fifteen year period and association with socio-demographic factors.

**Methods:**

We identified 4700 patients in the Health Improvement Network (THIN) primary care database, who had received treatment for bipolar disorder between 1995 and 2009. The proportion of time for which each individual was prescribed a particular medication was studied, along with variation by sex, age and social depravation status (quintiles of Townsend scores). The number of drugs an individual was taking within a particular year was also examined.

**Results:**

In 1995, 40.6% of patients with bipolar disorder were prescribed a psychotropic medication at least twice. By 2009 this had increased to 78.5% of patients. Valproate registered with the greatest increase in use (22.7%) followed by olanzapine (15.7%) and quetiapine (9.9%). There were differences by age and sex; with young (18–30 year old) women having the biggest increase in proportion of time on medication. There were no differences by social deprivation status. By 2009, 34.2% of women of childbearing age were treated with valproate.

**Conclusions:**

Lithium use overall remained relatively constant, whilst second generation antipsychotic and valproate use increased dramatically. Changes in prescribing practice preceded published trial evidence, especially with the use of second generation antipsychotics, perhaps with inferences being made from treatment of schizophrenia and use of first generation antipsychotics. Women of childbearing age were prescribed valproate frequently, against best advice.

## Introduction

Bipolar affective disorder is one of the commonest causes of disability worldwide, especially within the 15–44 age group [1]. The disorder usually emerges in adolescence or early adulthood, its most severe form is equally distributed between sexes [2], and typically follows an unpredictable course. Most treatment guidelines attempt to inform complex treatment decisions based on clinical trial findings. In clinical practice, however, patients are seldom as straightforward as those recruited to trials, in terms of illness characteristics, diagnostic heterogeneity, labile symptomatic presentations of the illness, and comorbidity [3]. Whereas most treatment trials have duration of months, the management of bipolar disorder is a lifelong effort to reduce symptoms and maximize quality of life.

Commonly used medications for maintenance treatment of bipolar disorder are mood stabilisers including lithium and anticonvulsants (valproate, carbamazepine, lamotrigine), first generation antipsychotics (FGAs), such as chlorpromazine and haloperidol, and second generation antipsychotics (SGAs), such as olanzapine and quetiapine.

There are limited data on the current prescribing patterns in patients with bipolar disorder in the UK. The evidence for best maintenance treatment of bipolar disorder is changing and conflicting [1], [4], [5], [6], and previous studies have shown substantial variation in prescribing practices [7], [8]. Maintenance treatments recommended by NICE [4] (which is generally recognized as producing the gold standard in prescribing guidelines) are lithium, valproate or olanzapine, and they recommend prescribing more than one of these medications if mood stabilization is poor. There are historical data from the 1990s suggesting that prescription patterns changed dramatically prior to publication of recent randomized controlled trials and guidelines, with an increase in the prescription of valproate, carbamazepine and lamotrigine, and a decrease in the use of lithium during the late 1990s [9], [10].

Most medication provided to patients with bipolar disorder in the UK is prescribed by general practitioners, following advice from secondary services. This has been the case since the mid-1990s when prescribing budgets were allocated to primary care services [11]. Therefore we planned to examine prescribing trends in bipolar disorder over the period 1995 to 2009, by using data gathered from primary care, which should accurately report prescribing trends in patients with the disorder.

The aim of this study was to examine changes in prescribing in the UK for bipolar disorder since the mid 1990s, and to identify socio-demographic predictors of these prescribing changes. We hypothesised that commonly used treatment regimens may not correspond with guidelines produced by NICE [4] or other advisory bodies [1], [5], [6].

## Methods

### Study design and setting

We carried out a retrospective cohort study of individuals in primary care with a diagnosis of bipolar disorder using The Health Improvement Network (THIN) primary care database.

### Data Source

THIN is one of the largest sources of primary care data in the United Kingdom containing information from over 470 general practices (accounting for over 9 million patients)(www.epic-uk.org). In the UK most people with severe mental illness are registered with primary care [12] and the validity of general practice computer diagnoses of severe mental illness has been established previously [13]. The database is broadly representative of UK general practice consultations and prescribing statistics [14]. THIN contains records of each patient's medical conditions and symptoms, recorded during routine consultations and all prescriptions issued by GPs. Symptoms and diagnoses are classified using the Read code system, a hierarchical recording system used to record clinical summary information [15]. This creates a computerized medical history for each patient from the time they register with a general practice. For this study patients were only included after the date on which their practice reached an acceptable standard of data recording [16]. In addition, the database holds information on basic demographics and social deprivation (measured using quintiles of Townsend score). The Townsend score is based on a patient's postcode, linked to population census data from 2001 [17]. It is a combined measure of owner-occupation, car ownership, overcrowding and unemployment [18].

All diagnoses of bipolar disorder were identified by Read codes in the patient's clinical records. Oral mood stabilisers and antipsychotics prescribed in primary care were identified based on encrypted multilex codes mapped to the British National Formulary. Both the list of Read codes and drug codes were created using the method described by Dave & Peterson [19].

The THIN scheme for obtaining and providing anonymous patient data to researchers was approved by the National Health Service South-East Multicenter Research Ethics Committee (MREC) in 2002. The current study was reviewed and approved by theLondon Research Ethics Committee, reference number: 09/H0718/11.

### Participants

Eligible patients were defined as being 18 years or over with at least one recorded diagnosis of bipolar disorder between 1995 and 2009. Patients were excluded if they had a diagnosis of epilepsy (identified by Read codes in the patients records), because of the overlap in prescribing of antiepileptic medications as mood stabilisers.

### Statistical Methods

To explore time trends in prescriptions we first identified individuals with two or more prescriptions of oral antipsychotic or mood stabiliser medication, as this would suggest a physician's intention to treat with a particular medication and initial patient concordance. We then analyzed the frequency of prescriptions by calendar year. We assessed the proportion of time for which each individual was prescribed a particular medication. This time was organized into treatment sessions, defined as a period of follow-up within which drug prescribing was continuous. As has been suggested previously [20], [21] we defined the end of a treatment session as a gap of 3 months or more between subsequent prescriptions. To aid analysis treatment sessions were defined in 3 levels: Level 1 – any antipsychotic prescription or any mood stabiliser prescription, Level 2 – class of treatment namely first generation antipsychotic (FGA), second generation antipsychotic (SGA), anticonvulsant (carbamazepine, lamotrigine, valproate), or lithium, Level 3 – individual antipsychotic or mood stabiliser medications. The data were then stratified by sex, age-group and social deprivation to assess whether each of these variables influenced any differences in treatment, or time spent in treatment. Particular attention was paid to subgroups that have been identified in guidelines as having specific needs/risks associated with treatment, such as women of childbearing age. To examine co-prescribing we studied the number of patients in a year issued two or more prescriptions for two or more psychotropics. All analysis was conducted using Stata version 11 for Windows.

## Results

### Sample demographics

There were 5,224 patients diagnosed with bipolar disorder (2,017 men, 3207 women), 4,700 (90.0%) of whom received at least two concurrent prescriptions of an oral antipsychotic or mood stabiliser during the study period. The sample demographics of the treated and untreated (issued less than two prescriptions) groups are described in Table 1.

**Table 1:** Demographics of the treated and untreated groups.

	Treated	Untreated (<2 prescriptions)
N	4700	524
Men (%)	1795 (38)	230 (44)
Mean age (S.D)	44.5 (15.2)	39.7 (15.6)
Median Follow-up (IQR)	7.69 (4.5–10.0)	4.98 (2.4–8.3)
Townsend Score (%)		
1 (least deprived)	859 (19)	108 (21)
2	797 (18)	100 (20)
3	983 (22)	99 (19)
4	1025 (23)	104 (20)
5 (most deprived)	830 (18)	98 (19)

### Overall trends

In 1995, 39/96 (40.6%) of patients with bipolar disorder were prescribed a psychotropic medication at least twice. By 2009 this had increased to 3037/3870 (78.5%) of patients. The proportion of individuals prescribed a medication at least twice rose by a mean of 2.7% per year.

Table 2 shows the changes in proportion of time spent on antipsychotic or mood stabiliser medication between 1995 and 2009, by sex, age band and Townsend score.

**Table 2:** Time spent in treatment with antipsychotic and mood stabiliser medication by sex, age group and Townsend score.

ALL ANTIPSYCHOTICS						
	1995		2009		Difference	Proportion
	%	Total person-years	%	Total person-years	2009-1995 (%)	2009/1995
Gender						
Male	19.8	9.6	38.6	1275.6	18.8	1.9
Female	11.2	18.5	41.9	2056.7	30.7	3.7
Age						
18–29	0	3.3	34	239.6	34	*
30–44	17	9.2	38.7	987	21.7	2.3
45–59	16.2	9.2	42.2	1223.9	25.9	2.6
60–75	14.6	6.4	42.5	881.7	27.9	2.9
Deprivation						
1 (Least deprived)	11.6	4.7	34.7	621.1	23.1	3
2	9.5	4.1	35.4	595.2	25.9	3.7
3	14.1	4.2	38.6	708.7	24.5	2.7
4	9.8	7.4	45.9	708.9	36	4.7
5 (Most deprived)	24.9	4.0	49.2	568.4	24.2	2

ALL MOOD STABILISERS						
	1995		2009		Difference	Proportion
	%	Total person-years	%	Total person-years	2009-1995 (%)	2009/1995
Gender						
Male	33.1	9.6	57.2	1275.6	24.1	1.7
Female	24.7	18.5	57.7	2056.7	33	2.3
Age						
18–29	8.6	3.3	40.9	239.6	32.2	4.7
30–44	22.9	9.2	52.8	987	29.9	2.3
45–59	29.4	9.2	61.8	1223.9	32.4	2.1
60–75	41.4	6.4	61.3	881.7	19.9	1.5
Deprivation						
1 (Least deprived)	23.5	4.7	57.5	621.1	34.1	2.5
2	38.3	4.1	59.4	595.2	21.2	1.6
3	8.6	4.2	56.4	708.7	47.8	6.6
4	40.6	7.4	57.4	708.9	16.8	1.4
5 (Most deprived)	14.5	4.0	56.4	568.4	41.9	3.9

**Figure 1:**
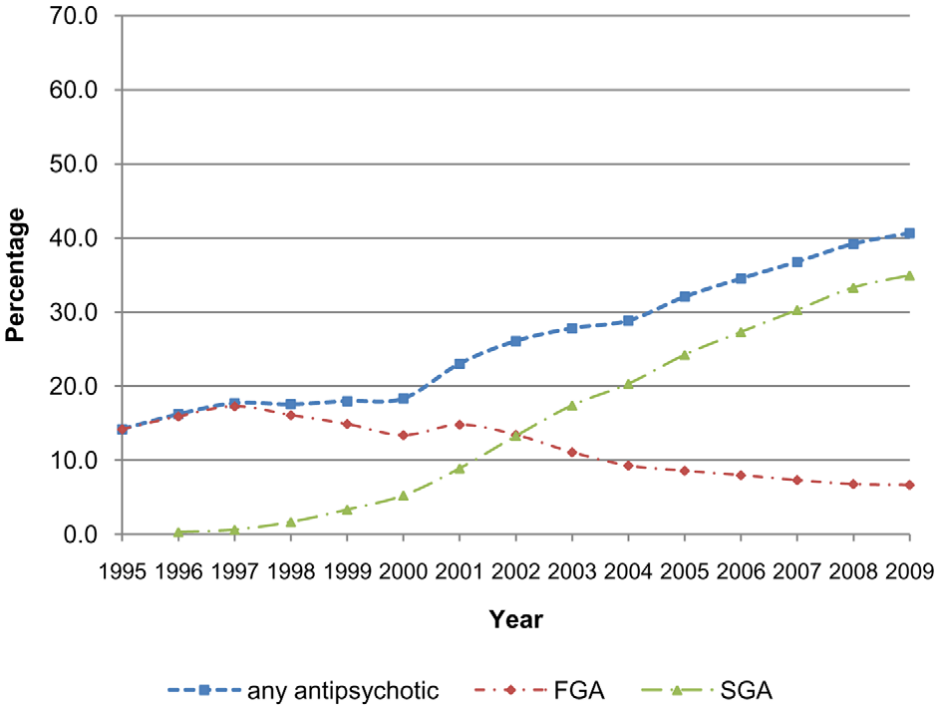
Proportion of time in treatment with antipsychotic medication.

**Figure 2:**
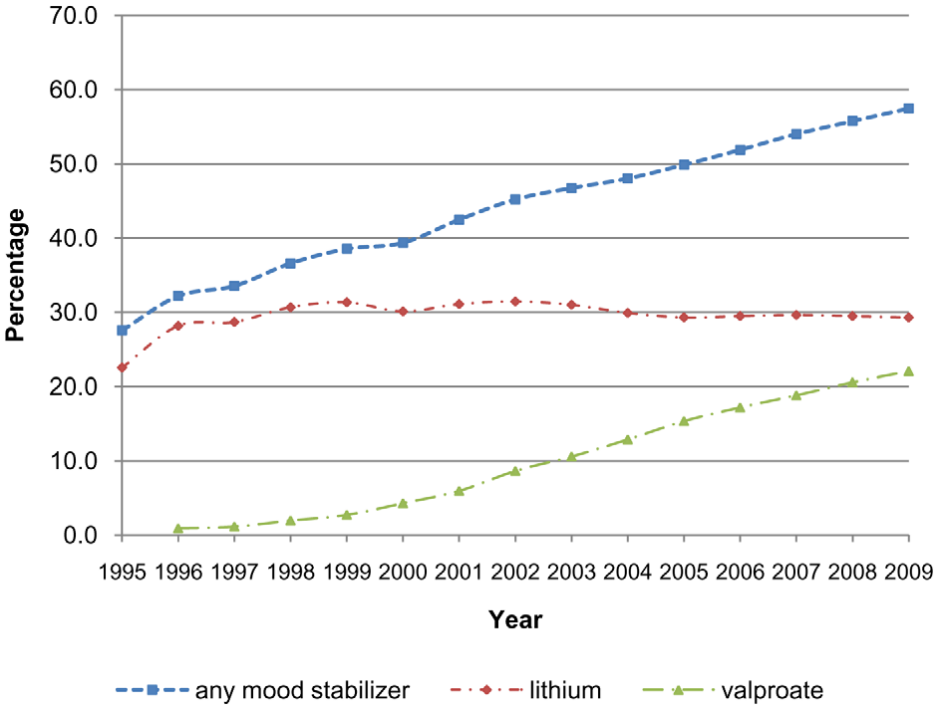
Proportion of time in treatment with mood stabiliser medication.

There was a 26.4% increase in the overall proportion of time spent on any antipsychotic medication between 1995 (14.2%) and 2009 (40.6%) (Figure 1), and a 29.9% increase in the proportion of time spent on any mood stabiliser over the same time period (27.5% to 57.4%) (Figure 2). This increase was larger for women for both types of medication (antipsychotics; 18.7% increase for men, 30.7% increase for women, mood stabilisers; 24.1% increase for men 33.0% increase for women). Some of this difference may be explained by the rapid increase in proportion of time spent on medication between 1995 and 1996 in the female group. However excluding the 1995 data still shows a larger increase for females. The biggest increase in proportion of time spent on both antipsychotic medications and mood stabilisers was in the 18–30 age range (34.0% and 32.2% respectively over the study period). There was no apparent difference between prescribing of antipsychotics or mood stabilisers by Townsend score (Table 2).

### Antipsychotics

In 1995 the proportion of time patients spent on first generation antipsychotics was 14.2%. By 2009 this had reduced to 6.9%. In contrast, the proportion of time spent on second generation antipsychotics had increased from zero to 35.0% (Figure 1).

**Figure 3:**
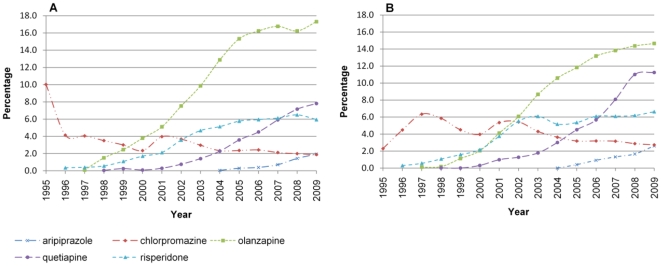
Prescribing of the 5 most common antipsychotic medications by sex. A) Male, B) Female.

The most commonly prescribed antipsychotics used for bipolar disorder in 1995 were 1) chlorpromazine, 2) haloperidol and 3) trifluoperazine. In 2009 most popular were 1) olanzapine 2) quetiapine 3) risperidone (Figure 3). Older people (60–75 age range) spent a greater proportion of time on olanzapine than those in younger age groups (18.9% in 2009, compared to 12.4% for 18–30 year olds). This was not observed for other antipsychotic medications and was reversed for aripiprazole, with patients aged 18–30 years spending more time in treatment (4.3% in 2009 compared to 1.0% in 2009). Men were more likely to spend time in treatment with olanzapine than women in 2009 (17.3% vs. 14.6%), where as women were more likely to spend time in treatment with quetiapine than men (11.3% vs. 7.8%) (Figure 3). There were no apparent differences by Townsend score.

### Mood stabilisers

**Figure 4:**
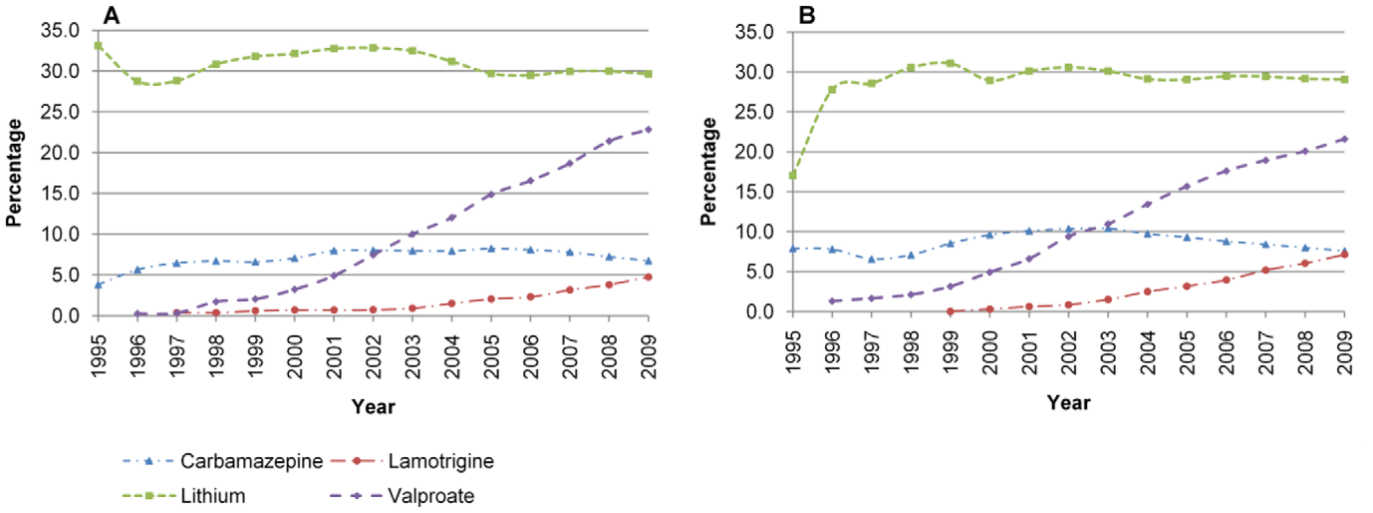
Prescribing of mood stabilisers by sex. A) Male and B) Female.

**Figure 5:**
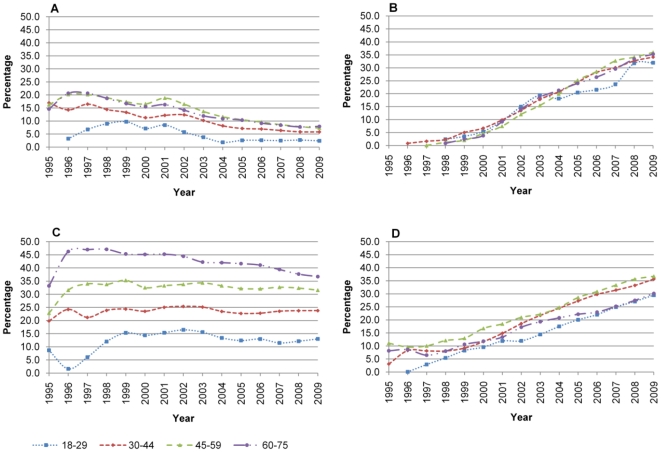
Prescribing by age group. A) First generation antipsychotic, B) Second generation antipsychotic, C) Lithium, D) Anticonvulsant.

Use of lithium increased from 22.5% in 1995 to 29.3% in 2009 (Figure 2). Over the same period valproate use increased from zero to 22.7%. The proportion of time men spent on lithium reduced by 3.5% (33.1% in 1995 to 29.6% in 2009), whereas for women the proportion increased by 12.1% (17.0% to 29.1%). For valproate the proportion of time spent in treatment increased by 22.8% (0% to 22.8%) and 21.6% (0% to 21.6%) for men and women respectively (Figure 4). The proportion of time spent on lithium was greater for older patients, and this was consistent throughout the study period, such that the mean proportion of time spent on lithium of different age groups was: 18–29, 12.1%; 30–44, 23.5%; 45–59, 32.4%; 60–75, 42.3% (Figure 5). This trend was more pronounced for women than men, but was present in both sexes. There were no apparent differences by Townsend score.

In 1995 none of the women of childbearing age (18–45 years old) in our sample were prescribed valproate. By 2009, 233 out of the 682 women with two or more prescriptions that year were taking valproate (34.2%) and spent 35.6% of the year in treatment.

Time spent in treatment with carbamazepine increased from 6.5% in 1995 to a peak of 9.5% in 2004; by 2009 this had reduced to 7.3%. The proportion of time spent in treatment with lamotrigine increased from zero to 6.2% (Figure 4). Neither of these drugs showed differences by sex, age or Townsend score.

### Co-prescribing

**Figure 6:**
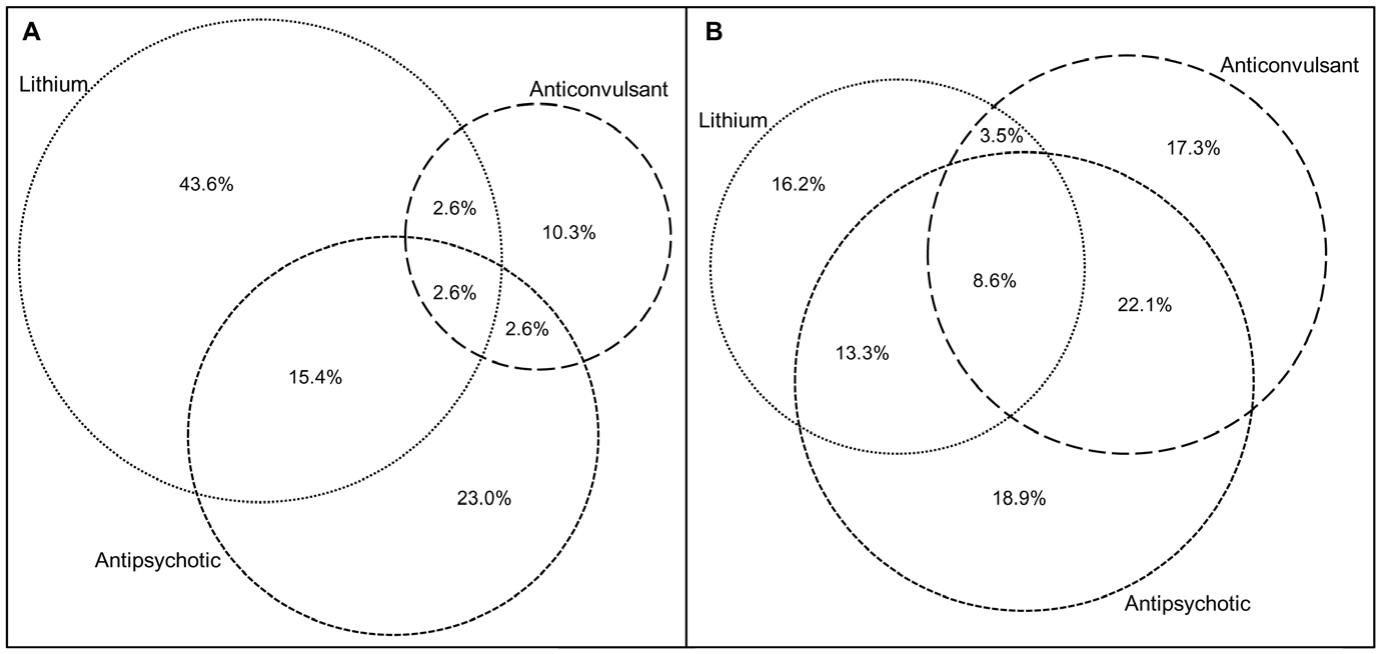
Percentage of treated individuals by medication group in A) 1995 and B) 2009*. *Not to scale - 1995 euler diagram should be approximately 1/80^th^ the size of 2009.

In 1995, 9 out of 39 individuals (23.1%) were issued two or more prescriptions for more than one psychotropic medication; by 2009 this had increased to 1,461 out of 3,037 (48.1%). In 1995, 7 (17.9%) patients were prescribed lithium and an antipsychotic, which were all FGAs. In 2009, 665 (21.9%) individuals were prescribed lithium and a FGA or SGA. In 1995, 2 (5.2%) patients were prescribed an anticonvulsant (valproate, carbamazepine or lamotrigine) and an antipsychotic; by 2009 this had increased to 932 (30.7%). Lithium and an anticonvulsant was prescribed to 5.2% of the population (2/39) in 1995, by 2009 this stood at 12.1% (367/3037) (Figure 6).

Over the 15 years of the study, patients prescribed lithium plus an antipsychotic tended to spend an approximately equal proportion of time on antipsychotic medication (mean 23.5%) as those prescribed valproate plus an antipsychotic (mean 25.7%). However those on lithium plus an antipsychotic spent a higher proportion of time taking lithium than the valproate plus antipsychotic cohort spent taking valproate (means; 60.8% vs. 29.8%).

## Discussion

Our results indicate that from 1995 to 2009 prescribing, broadly speaking, corresponded with availability, licensing and guidelines. Key findings are: 1) the proportion of patients offered treatment for bipolar disorder increased markedly between 1995 and 2009; 2) patients spent ever increasing amounts of time on psychotropic medication, in particular second generation antipsychotics and valproate; 3) this increase in time on medication was most noticeable in younger women; 4) antipsychotic and valproate prescribing increased relative to lithium; 5) use of second generation antipsychotics accelerated; 6) prescribing more than one drug at once increased; 7) treatment was not influenced by social deprivation and; 8) by 2009 one third of women of childbearing age who took medication for bipolar disorder were taking valproate. This final finding is worrying as guidelines are now very clear that valproate should be avoided because of its teratogenic potential.

The long-term management of bipolar disorder is complex. The prescribing recommendations described in the 2006 NICE guidelines [4] should represent the gold standard, but they are unusually vague and recently a review and update has been requested [22]. NICE recommends lithium, olanzapine or valproate as first line, which we have found to be the three most commonly prescribed psychotropic medications for maintenance. If the patient has frequent relapses, or symptoms continue to cause functional impairment, they recommend switching to an alternative monotherapy or adding a second prophylactic agent (lithium, olanzapine, valproate). However the evidence supporting the maintenance use of olanzapine and valproate is limited, and valproate is not licensed for this use. If anything, the evidence from research carried out after 2006 further strengthens the argument for use of lithium first line [1], [23], [24], [25], but the complications of initiation, monitoring and side effect profile may continue to limit its use. It is also recognised that irregular use of lithium produces poor outcomes [26], risk of relapse on stopping [27] and that less than two years use may have no beneficial effect [28]. It may therefore be that the degree of concordance suggested by our findings reduces its benefit relative to other maintenance medications.

The increase in time spent on medication is likely to represent both increased prescribing and increased adherence to medication [20], [29]. Previous studies of antipsychotic prescribing trends have found that, over time, patients have been prescribed medications (for all indications) for longer periods [30], and this has been shown specifically in the bipolar disorder patient group [31]. Our results differ from some studies from the United States, which found that lithium prescription for bipolar declined, over the period 1990–2005 [31], [32], [33]. The latter two studies also failed to show the increase in antipsychotic prescriptions found in this study.

It appears that use of second generation antipsychotics for bipolar disorder pre-empted the available scientific evidence. The first case reports suggesting the effective treatment of mania with olanzapine were published in 1997 [34], [35]. These were followed by the publication of the first randomised control trials in 1999 [36], [37]. Olanzapine was first given marketing authorisation for psychosis in 1996, and was first used in our cohort in 1997. Risperidone was first used in our study in 1996, two years before the first published randomised control trial into its effectiveness as monotherapy [38], but four years after it was first authorised. Quetiapine, authorised for schizophrenia initially in 1997, was first used in our sample in 1998, while the first trial of quetiapine as an add-on medication was not published until 2004 [39]. However it does seem reasonable that clinicians made inferences from trials of second generation antipsychotics in psychosis and clinical experience of first generation antipsychotics.

Concerns were raised about the teratogenic effects of valproate in the early 1980s [40], but it was only in 2004 that the risk was confirmed to be higher than other anticonvulsants [41], [42]. Therefore psychiatrists prescribing in this population may only have become aware of this risk via the NICE guidelines from 2006 onwards [4]. However, despite this advice, use of valproate has continued to rise since then.

Previous studies have shown that co-prescribing is common [43], [44] with up to 80% of patients on a mood stabiliser plus another medication. In our cohort 48.1% were prescribed more than one agent in 2009, and an anticonvulsant plus an antipsychotic became the most commonly used dual therapy. Co-prescribing, although concordant with guidelines is problematic due to the side effect profiles of the drugs used, and concerns over long term health risks [1].

### Limitations

THIN is a primary care database and, as with all clinical databases, it is impossible to be sure that a person prescribed a psychotropic medication was concordant. However, it is fair to assume that repeat prescription of a particular drug implies medication collection, from which we may infer some degree of adherence. Gaps in treatment may be explained by hospital prescribing, such as during acute inpatient stays, and therefore we may be underestimating the duration of treatment. Also, there may be a number of patients who receive all their medication from secondary care, though these numbers are likely to be small, given the manner in which prescribing budgets are allocated in the UK. Secondary care prescribing may have been higher earlier in the study time frame, but analyzing the results excluding the first 5 years of data does not change the findings. There is no reason to suppose that any particular group would have been preferentially prescribed for in secondary rather than primary care. Although the focus of this study is primary care prescribing, specialist treatment is likely to be a major influence on GP prescribing, and so it is likely that these trends would be reflected in the total population with bipolar disorder.

We were unable to separate bipolar I disorder from bipolar II disorder in our cohort; however given that treatment guidelines are the same for both subtypes of the disorder (extrapolated from research in bipolar I), the trends in prescribing are likely to be very similar. Changes in diagnostic practice probably mean there was an increase in the number of bipolar II patients over the study period. From our current study we are unable to comment on whether a drug was prescribed initially as a monotherapy or, if not, in what order it may have been added to the treatment regimen. We also do not know about historical prescribing for the cohort so we are unable to state whether clinicians have adhered to the guidelines for first line drugs on initiation of treatment. 62% of the treated sample were women, although the overall incidence in men and women is thought to be equal, it is recognised that females have more acute episodes of illness [45], and therefore their information may be better recorded in the database. Our cohort was also relatively old at the start of follow-up (mean 44.5 years) compared to the observed age of onset of the disorder, which tends to be in the early twenties [45]. It is likely therefore that some our sample is previously diagnosed cases that have entered the database late. It may be that trends in treatment of newly diagnosed individuals (incident cases) differ from our findings.

### Conclusions

This study identified broad concordance with prescribing guidelines. Our findings suggest a number of important trends that should be noted by researchers and clinicians alike, the most striking being the overall increase in prescribing and time spent in treatment. A number of questions remain unanswered about the long-term management of bipolar disorder. Although there is unlikely to be one ideal treatment for all patients with bipolar disorder, as the illness is heterogeneous and subtypes appear to be associated with a preferential response to specific drugs [46], further studies with long follow-up times are necessary to clarify the benefits (and risks) of different psychotropic medications, especially antipsychotics. Despite this it would be useful to prescribers (both psychiatrists and GPs) if NICE guidelines were able to be more precise about recommendations, especially in the areas of first line treatments and treatments for women of childbearing age. Perhaps there also needs to be more education about these areas.
